# Extending Absorption Edge through the Hybrid Resonator-Based Absorber with Wideband and Near-Perfect Absorption in Visible Region

**DOI:** 10.3390/ma13061470

**Published:** 2020-03-24

**Authors:** Yen-Chuan Lai, Chia-Yuan Chen, Yun-Ting Hung, Chia-Yun Chen

**Affiliations:** 1 Department of Materials Science and Engineering, National Cheng Kung University, Tainan 70101, Taiwan; to50210ny@gmail.com (Y.-C.L.); pen1356time@gmail.com (Y.-T.H.); 2 Department of Mechanical Engineering, National Cheng Kung University, Tainan 70101, Taiwan; chiayuac@mail.ncku.edu.tw; 3 Hierarchical Green-Energy Materials (Hi-GEM) Research Center, National Cheng Kung University, Tainan 70101, Taiwan

**Keywords:** light absorber, optical metamaterials, resonances, broadband absorption

## Abstract

Metamaterial absorber with the unexpected capability for harvesting electromagnetic energy has been regarded as a potential route for various applications, including chemical/biological sensing, cloaking and photovoltaic applications. In this study, we presented the simple absorber design made with Al/SiO_2_/Al sandwich structures through the involvement of hybrid dual-resonators that could allow the wideband light absorption covered from 450 nm to 600 nm with average absorptivity above 95%. Examinations of excited electric field, magnetic field and total magnitude of electric field in three-dimensional space at resonances were performed to clarify the origin of resonant behaviors. In addition, an equivalent inductance–capacitance circuit model was proposed that could qualitatively explore the geometry-dependent absorption characteristics by modulating the constitutive parameters of hybrid resonators. As a result, the designed light absorber might enable to be practically applied for various optical-management and photovoltaic applications, and even offered the tunability for other desired frequency regions.

## 1. Introduction

In 2001, Smith et al., have experimentally demonstrated a man-made design, termed as metamaterials that has attracted substantial attention due to its unprecedented material characteristics and innovative applications such as invisible cloaking, superlens, optical sensing and photovoltaic [1]. It enabled to manipulate the electromagnetic transportation through the designable permittivity and permeability [2,3,4] based on the artificial structure design that featured the negative-refractive index and evanescent wave amplification which could not be fond in natural materials [5,6,7]. For instance, metamaterial absorber (MMA) has been designed to realize the more efficient use of electromagnetic waves, which possessed the unexpectedly high light-absorptivity by the impedance at the resonant spectral position of metamaterials matching the impedance of the free space that caused the reflected and transmitted waves turning to be nearly zero. The first report of MMA was proposed by Landy et al., [8], where the ultra-strong light absorption at microwave frequency was realized by the two distinct resonators separated with a dielectric substrate. In such design, the impedance, determined by the magnetic permeability and electric permittivity, could be potentially tailored through the resonator design, and thus was matched with that of surrounding media [9,10]. Until today, the correlated explorations of MMA structures were covered from microwave, infrared (IR) toward visible region by changing the structural dimension as well as geometries of involved resonators [11,12,13,14]. Tao et al., [15] proposed a flexible metamaterial absorber with magnetic resonance formed by the strong coupling effect between the dielectric layer and the metal layer, which demonstrated the nearly 100% of absorptance at 1.6 GHz. Li et al., [16] presented a metamaterial absorber with three absorption peaks in the gigahertz region, and the absorber was polarization independence because of the symmetrical design of the resonant structures. Besides, Ding et al., [17] designed an absorber that possessed the absorptance reaching 90% at frequencies of 7.8 GHz and 14.7 GHz, respectively, instead it owned the polarization dependency under the incident angles less than 60°.

For photovoltaic applications, the efficient way for trapping the visible lights within photoactive regions turned out to be essential for boosting the cell efficiency [18,19,20]. In such a requirement, the light-absorption characteristics essentially required the broadband and polarization-angle insensitive capabilities. The spectral regions of the design absorber should particularly suit for absorbing the light wavelength of 580 nm as it belonged to the strongest irradiance of solar energy. Taking together, the current work presented a new class of MMA designs while the designed structures were less complicated than the previously proposed architectures. Unlike the previous report where only the single or multiple absorption peaks could be realized [21,22,23], in this study, the employment of two sets of sandwich resonators in the form of Al/SiO_2_/Al sandwich structures enabled to come out the tailored electromagnetic resonances that benefited the realization of broadband and high-quality light-absorption capability covering from 450 nm to 600 nm with polarization insensitivity. Furthermore, we explored the contributions of the geometric dimensions in resonator designs in order to interpret their resonant mechanism for the involvement of achieving near-perfect and broadband absorptance.

## 2. Materials and Methods 

The designed MMA structures composed of sandwich structures, where, from the top to the bottom sides were the patterned Al resonators, SiO_2_ dielectric spacer and continuous Al layer directly adhered to the glass substrates. The correlated metallic resonators could be categorized into two sets of periodic designs, including square-shape resonators at four corners and strip-shape resonant structures at central regions. For exploring their artificial electromagnetic responses, Finite-Difference Time-Domain (FDTD) was employed with the Lumerical software (8.15.736, Lumerical, Vancouver, BC, Canada). The periodic boundary conditions along x-axis and y-axis were assigned, and the plane-wave excitation was along the z-axis. The refractive and extinction coefficient of the Al and SiO_2_ used in MMA design was originated from the literature [24]. Specifically, the wavelength-dispersive refractive index and extinction coefficient was set to be 0.5–2.4 and 4.9–8.62 for Al, and 1.47–1.45 and 0 for SiO_2_ in the wavelength range of 400–750 nm, respectively. Examinations of light absorbance (A(λ)) in MMA structures were mainly determined by two frequency-dependent parameters, which were transmittance (T(λ)) and reflectance (R(λ)) following the relation, A(λ) = 1 − T(λ) − R(λ). Since the thickness of Al metallic layer was much greater than the skin depth at a resonant frequency that the incident light penetrating though metallic layers could be ignored. Under such circumstances, the transmission was almost zero (T(λ) ≈ 0) due to the existence of a continuous Al layer, such that the absorbance could be evaluated by, A(λ) = 1 − R(λ).

## 3. Results and Discussion

**Figure 1:**
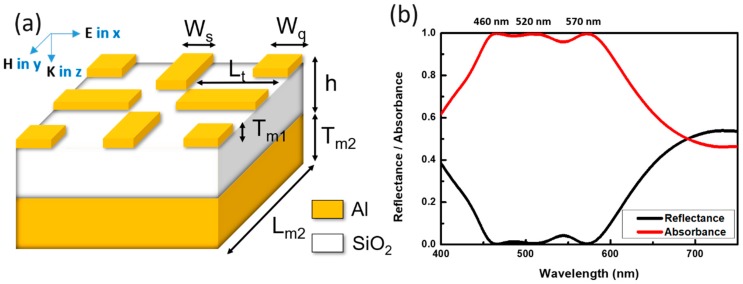
(**a**) Schematic illustration of the proposed dual-resonator based light absorber with defined structural parameters. (**b**) The calculated reflectance and absorbance spectra of the designed light absorber.

**Figure 2:**
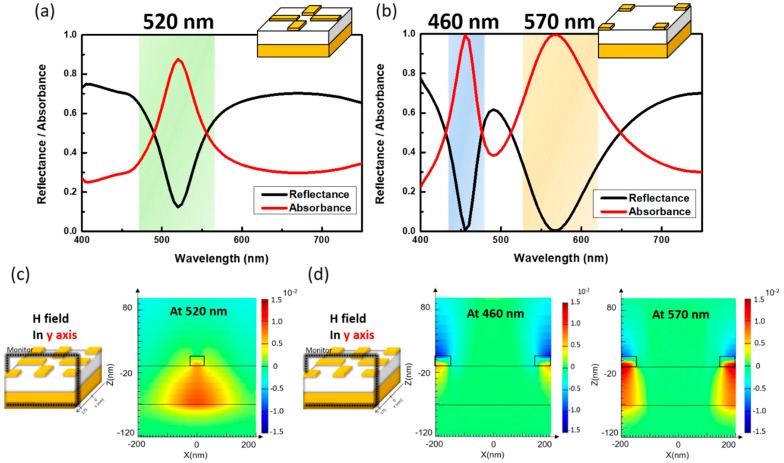
The calculated reflectance and absorbance spectra of (**a**) sole sandwich strip-like resonators and (**b**) sole sandwich square-like resonators. The corresponding evaluated magnetic field along the y-axis (**c**) at 520 nm from sandwich strip-like resonators and (**d**) at 460 nm and 570 nm from sandwich square-like resonators.

The metamaterial structures were illustrated in Figure 1a, where the architectures essentially consisted of two types of Al/SiO_2_/Al sandwich resonators in periodic form. Accordingly, the main geometric parameters were W_q_ = 50 nm, L_m2_ = 400 nm, L_t_ = 160 nm, T_m2_ = 50 nm, h = 65 nm, T_m1_ = 15 nm and W_s_ = 40 nm, and the metamaterial resonators were readily illuminated with light under normal incidence. Sınce the designed structures unambiguously possessed four-fold symmetry relative to the incident direction, the effect of polarization anisotropy could be ignored. The correlated optical reflectance and absorptance spectra could be found in Figure 1b. It indicated that the extraordinary broadband light absorption capability from such dual-resonator hybrid structures. Specifically, the absorption peaks could be found at 460 nm, 520 nm and 570 nm with nearly 100% absorptivity and the average absorptivity could reach above 95% covering from 450 nm to 600 nm. This implied that by well incorporating resonant behaviors from hybrid-resonator-based system it could respond to the strong and wideband light-harvesting effect. To further elucidate their light absorption characteristics, an attempt to feature their original resonant characteristics by separating the resonant structures, including strip-like structures at central region and square-like resonators at corner positions from the hybrid system was performed, as shown in Figure 2.

From sole strip-based resonators, the calculated spectra indicated that the strong absorption peak located at 520 nm within the examined wavelengths of 400 to 750 nm, as shown in Figure 2a. The correlated full width at half maximum (FWHM) and peak absorptivity were 53 nm and 90%, respectively, presenting the high quality in terms of resonant behavior. The corresponding magnetic-field distribution at resonance wavelength was depicted in Figure 2c for the understanding of their resonant characteristics, where the field monitor was located at x–z plane and intercepted with y-axis at 175 nm to examine the magnetic field in y-axis at resonance. It could be observed that the induced magnetic dipoles were strongly localized within the intermediate SiO_2_ region along the y-axis. This could be attributed to the fact that the antiparallel induced currents were excited at upper Al resonators and bottom Al layer with the result of magnetic dipoles at orientation opposite to the incoming magnetic component of the incident field, thus leading to the creation of magnetic resonance and in turn, reflecting the collective absorption of incoming electromagnetic energy at resonating. In addition, when considering the sole square-based resonators periodically located at the corners of unit cells, two distinct resonant peaks could be observed which were centered at 460 and 570 nm, respectively, as shown in Figure 2b. To further visualize the origin of such excited resonances, the examination of the induced magnetic field along y-axis was performed, as presented in Figure 2d. It indicated that at 460 nm no obvious magnetic dipoles were excited, reflecting that the pure dipolar resonant types instead of magnetic resonance were created. In such configuration, each square resonator behaved as electric dipoles that constituted the plasmonic resonances with strong absorption of light at a resonant wavelength of 460 nm. By contrast, the absorption peak at 570 nm stood for the distinct resonant characteristics, which essentially trapped the incoming light within the middle SiO_2_ medium of Al/SiO_2_/Al-based sandwich structures. As illustrated in Figure 2d, the strongly localized magnetic field was concentrated in between patterned Al resonators and a continuous Al layer that encountered the magnetic resonance in accordance with the resonant response from sandwich-based strip resonators, as depicted in Figure 2c, where the comparably smaller resonant wavelengths of square resonators could be associated with the correlated resonators with smaller dimension.

**Figure 3:**
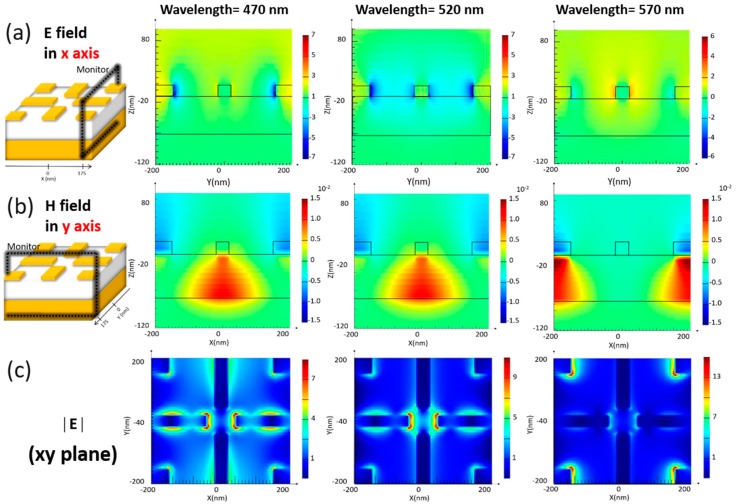
Examinations of (**a**) electric field in the x-axis, (**b**) magnetic field in the y-axis and (**c**) the total magnitude of electric field at resonances.

By integrating strip and square resonators as a hybrid sandwich system, the broadband absorption characteristics rather than three distinct absorption peaks could be created, as demonstrated in Figure 1b. The further interpretation could be supported by examining the electric field in x-axis, the magnetic field in y-axis and total magnitude of electric field in three-dimensional space, as compared in Figure 3. For examining the electric field in x-axis at resonance, the field monitor was configurated at yz plane and intercepted with x-axis at 175 nm, as shown in Figure 3a. It could be obtained that at 520 nm, the associated resonance was caused by the excitation of magnetic resonance from central strip resonators (Figure 3b), which caused the trapping of incoming light within the dielectric medium. Such effect was originally weakened in sole strip resonators at wavelengths away from 520 nm, instead in hybrid resonators, the strong light absorption characteristics were substantially extended toward the lower and higher wavelengths regions due to the incorporation of square resonators at corners of the hybrid system. Specifically, at a larger wavelength region, the additional illuminated magnetic resonance was also excited at 570 nm because of the magnetic-field localization from sandwiched square resonators, as presented in Figure 3b, thus leading to the absorption edge shifted toward 600 nm. At low wavelength region compared with the absorption center at 520 nm, the dipolar plasmonic resonance from square resonators and magnetic response from sandwich strip resonators were both excited and turned out to be strongly coupled. Such resonant coupling between two resonances was initiated, which could be clearly observed from the field mapping of the total electric field (Figure 3c) at the top view of hybrid resonators. This caused the effective wavelength-independent absorption behaviors at a cut-off wavelength near 450 nm, and overall resulted in the highly broadband light absorption features covering 450 nm to 600 nm.

**Figure 4:**
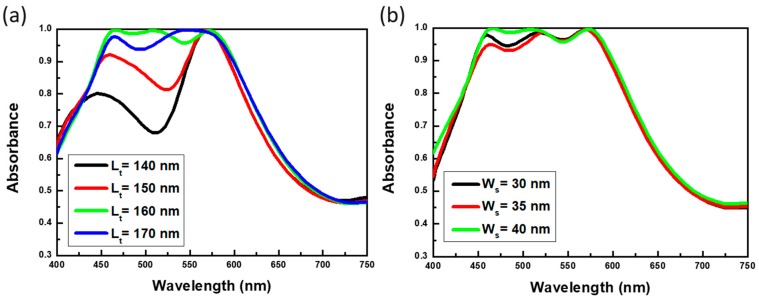
Contributions of varying the (**a**) strip lengths and (**b**) strip widths to the absorptance spectra.

**Figure 5:**
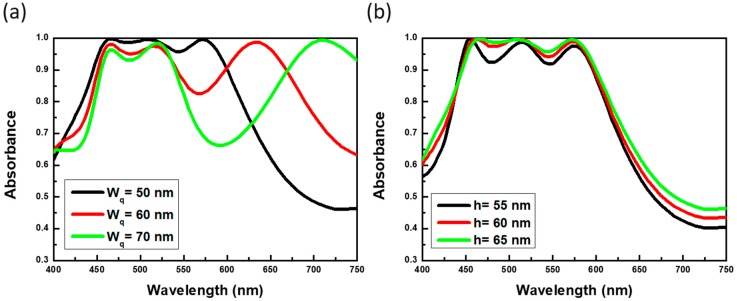
Contributions of varying the (**a**) square widths and (**b**) thickness of the dielectric layer to the absorptance spectra.

The involving resonant mechanism could be further understood by varying the structural dimensions of hybrid resonators, as compared in Figure 4 and Figure 5. An equivalent inductance-capacitance (LC) circuit model [25,26,27] was applied to qualitatively explore the geometry-dependent absorption characteristics. Notice that this model was also employed in near-IR [28] and visible region [29], respectively. In this aspect, the sandwich-based strip resonators could be regarded as effective LC resonators. Specifically, the inductance (L) of sandwich-based strip resonators could be approximately expressed with the formula, L~µ_0_L_t_(h/w_s_) by considering the form of discontinuous metallic wires, where µ presented the free space permeability and L_t_, W_s_ and h correlated with the resonator length, width and dielectric thickness, respectively. Besides, the involving capacitance (C) established by two plane-based capacitors could be described by the following expression, C~ε_r_ε_0_w_s_L_t_/4h, where ε_r_ and ε_0_ were the relative and free space permittivity, respectively. Furthermore, the corresponding LC resonant frequency (f_R_) was represented as f_R_ = $1/\left(2\pi \sqrt{\mathrm{LC}}\right)\;$= C_0_/${\pi L}_{t}\sqrt{\varepsilon_{r}}$, where C_0_ was the speed of light in the vacuum. The results indicated the fact that the resonant spectral position of strip resonators was solely dependent on the strip length and dielectric spacing between upper Al strips and Al layer at the bottom.

To clarify the present model, the lengths of metallic strips (L_t_) were modulated, as shown in Figure 4a. It evidenced that the resonant wavelengths were red-shifting with the increase of strip length, and were eventually merged with the third resonant position (570 nm). Such results corresponded with effective LC circuit model, where the resonant frequency negatively correlated with the strip length of upper Al strip resonators. In addition, the third resonant peaks at 570 nm stayed consistent regardless of the length variations, which identified only the second resonance at 520 nm readily related to the resonant behavior of strip resonators, whereas the resonance at 570 nm was correlated with the resonant behavior of square resonators. Moreover, Figure 4b displayed that both the second and third resonant peaks (520 nm and 570 nm) remained at original spectral positions while the strip widths were varied, and again the results matched with the prediction of LC circuit expression. By enlarging the widths of square resonators, it could be found that the third resonant peak at 570 nm shifted to the longer wavelength, as shown in Figure 5a. According to the LC circuit model where the resonant frequency was negatively correlative with the square width ($W_{q}$), i.e., f_R_ = $1/\left(2\pi \sqrt{\mathrm{LC}}\right)\;$= C_0_/${\pi W}_{q}\sqrt{\varepsilon_{r}}$, such that the LC-circuit concept was still valid for the involved resonant behavior of sandwich-based square resonators. Finally, the thickness of intermediate SiO_2_ layer was modified, and we could also observe the blueshift of spectral positions in both second (from sandwich-based strip resonators) and third (from sandwich-based square resonators) resonant positions, which could be also interpreted well with the LC circuit model, as presented in Figure 5b. Thus, the wideband light absorber could be realized through the optimized structure management associated with the LC circuit design, which could be tunable for other desired frequency regions. It has been reported that the structures with metal pattern/dielectric layer/metal layer could proceeded the excellent photocatalytic oxidation of benzylamine [30]. In light of this finding, the present study unveiled the near-perfect and broadband light-absorption characteristics based on the simple sandwich-like design, and could be potentially used for efficient photocatalytic applications illuminated with visible light through replacing the middle SiO_2_ layer with photochemical-active materials such as TiO_2_ [30] or Cu_2_O [31], which might pave ways for organic synthesis, pollutant degradation and other photocatalytic applications.

## 4. Conclusions

In summary, we demonstrated a broadband metamaterial-based absorber possessing polarization independence which could dramatically absorb the wideband visible lights through the integration of the dual-resonator hybrid system. By examining the geometric dimensions of designed resonators, the effective LC circuit model was visualized that could allow the comprehensive understanding of involved resonant behaviors, and such simple designs further allowed the highly enhanced light absorption covering the visible wavelength regions from 450 nm to 600 nm with average absorptance above 95%. These tailored wideband absorption characteristics featuring the excellent capability for harvesting sunlight could readily benefit the advanced development of solar cells, photocatalysis, sensing and other functional optoelectronic devices.

## References

[1] Smith D.R., Padilla W.J., Vier D.C., Nemat-Nasser S.C., Schultz S. (2000). Composite medium with simultaneously negative permeability and permittivity. Phys. Rev. Lett..

[2] Engheta N., Ziolkowski R.W. (2005). A positive future for double-negative metamaterials. IEEE Trans. Microw. Theory Tech..

[3] Engheta N. (2002). An idea for thin subwavelength cavity resonators using metamaterials with negative permittivity and permeability. IEEE Antennas Wirel. Propag. Lett..

[4] Smith D.R., Schultz S., Markoš P., Soukoulis C.M. (2002). Determination of effective permittivity and permeability of metamaterials from reflection and transmission coefficients. Phys. Rev. B.

[5] Shelby R.A., Smith D.R., Schultz S. (2001). Experimental verification of a negative index of refraction. Science.

[6] Smith D.R., Pendry J.B., Wiltshire M.C. (2004). Metamaterials and negative refractive index. Science.

[7] Valentine J., Zhang S., Zentgraf T., Ulin-Avila E., Genov D.A., Bartal G., Zhang X. (2008). Three-dimensional optical metamaterial with a negative refractive index. Nature.

[8] Landy N.I., Sajuyigbe S., Mock J.J., Smith D.R., Padilla W.J. (2008). Perfect metamaterial absorber. Phys. Rev. Lett..

[9] Avitzour Y., Urzhumov Y.A., Shvets G. (2009). Wide-angle infrared absorber based on a negative-index plasmonic metamaterial. Phys. Rev. B.

[10] Zhang S., Fan W., Malloy K.J., Brueck S.R., Panoiu N.C., Osgood R.M. (2006). Demonstration of metal-dielectric negative-index metamaterials with improved performance at optical frequencies. JOSA B.

[11] Hedayati M.K., Javaherirahim M., Mozooni B., Abdelaziz R., Tavassolizadeh A., Chakravadhanula V.S.K., Elbahri M. (2011). Design of a perfect black absorber at visible frequencies using plasmonic metamaterials. Adv. Mater..

[12] Liu N., Mesch M., Weiss T., Hentschel M., Giessen H. (2010). Infrared perfect absorber and its application as plasmonic sensor. Nano Lett..

[13] Liu S., Chen H., Cui T.J. (2015). A broadband terahertz absorber using multi-layer stacked bars. Appl. Phys. Lett..

[14] Shen X., Cui T.J., Zhao J., Ma H.F., Jiang W.X., Li H. (2011). Polarization-independent wide-angle triple-band metamaterial absorber. Opt. Express.

[15] Tao H., Bingham C.M., Strikwerda A.C., Pilon D., Shrekenhamer D., Landy N.I., Averitt R.D. (2008). Highly flexible wide angle of incidence terahertz metamaterial absorber: Design, fabrication, and characterization. Phys. Rev. B.

[16] Li H., Yuan L.H., Zhou B., Shen X.P., Cheng Q., Cui T.J. (2011). Ultrathin multiband gigahertz metamaterial absorbers. J. Appl. Phys..

[17] Ding F., Cui Y., Ge X., Jin Y., He S. (2012). Ultra-broadband microwave metamaterial absorber. Appl. Phys. Lett..

[18] Chen C.Y., Wei T.C., Hsiao P.H., Hung C.H. (2019). Vanadium oxide as transparent carrier-selective layer in silicon hybrid solar cells promoting photovoltaic performances. ACS Appl. Energy Mater..

[19] Putra I.R., Li J.Y., Chen C.Y. (2019). 18.78% hierarchical black silicon solar cells achieved with the balance of light-trapping and interfacial contact. Appl. Surf. Sci..

[20] Chen C.Y., Wei T.C., Lai Y.C., Lee T.C. (2020). Passivating silicon-based hybrid solar cells through tuning PbI2 content of perovskite coatings. Appl. Surf. Sci..

[21] Tao H., Landy N.I., Bingham C.M., Zhang X., Averitt R.D., Padilla W.J. (2008). A metamaterial absorber for the terahertz regime: Design, fabrication and characterization. Opt. Express.

[22] Ma Y., Chen Q., Grant J., Saha S.C., Khalid A., Cumming D.R. (2011). A terahertz polarization insensitive dual band metamaterial absorber. Opt. Lett..

[23] Wen Q.Y., Zhang H.W., Xie Y.S., Yang Q.H., Liu Y.L. (2009). Dual band terahertz metamaterial absorber: Design, fabrication, and characterization. Appl. Phys. Lett..

[24] Chan G.H., Zhao J., Schatz G.C., Van Duyne R.P. (2008). Localized surface plasmon resonance spectroscopy of triangular aluminum nanoparticles. J. Phys. Chem. C.

[25] Pendry J.B., Holden A.J., Robbins D.J., Stewart W.J. (1999). Magnetism from conductors and enhanced nonlinear phenomena. IEEE Trans. Microw. Theory Tech..

[26] Zhou J., Zhang L., Tuttle G., Koschny T., Soukoulis C.M. (2006). Negative index materials using simple short wire pairs. Phys. Rev. B.

[27] Chen C.Y., Chen C.Y., Hsiao P.H., Hsu C.C., Mani K. (2015). Efficient metamaterial-based plasmonic sensors for micromixing evaluation. J. Phys. D Appl. Phys..

[28] Linden S., Enkrich C., Wegener M., Zhou J., Koschny T., Soukoulis C.M. (2004). Magnetic response of metamaterials at 100 terahertz. Science.

[29] Lahiri B., McMeekin S.G., Khokhar A.Z., De La Rue R.M., Johnson N.P. (2010). Magnetic response of split ring resonators (SRRs) at visible frequencies. Opt. Express.

[30] Xiao Q., Connel T.U., Cadusch J.J., Roberts A., Chesman A.S.R., Gómez D.E. (2018). Hot-carrier organic synthesis via the near-perfect absorption of light. ACS Catal..

[31] Hsiao P.H., Li T.C., Chen C.Y. (2019). ZnO/Cu2O/Si nanowire arrays as ternary heterostructure-based photocatalysts with enhanced photodegradation performances. Nanoscale Res. Lett..

